# Changes in Cerebral Blood Flow during Olfactory Stimulation in Patients with Multiple Chemical Sensitivity: A Multi-Channel Near-Infrared Spectroscopic Study

**DOI:** 10.1371/journal.pone.0080567

**Published:** 2013-11-21

**Authors:** Kenichi Azuma, Iwao Uchiyama, Hirohisa Takano, Mari Tanigawa, Michiyo Azuma, Ikuko Bamba, Toshikazu Yoshikawa

**Affiliations:** 1 Department of Environmental Medicine and Behavioral Science, Kinki University Faculty of Medicine, Osakasayama, Osaka, Japan; 2 Sick-house Medical Science Laboratory, Division of Basic Research, Louis Pasteur Center for Medical Research, Kyoto, Japan; 3 Outpatient Department of Sick-house Syndrome, Hyakumanben Clinic, Kyoto, Japan; 4 Environmental Health Division, Department of Environmental Engineering, Graduate School of Engineering, Kyoto University, Kyoto, Japan; 5 Clinical Immune Function Laboratory, Division of Basic Research, Louis Pasteur Center for Medical Research, Kyoto, Japan; 6 Division of Internal Medicine, Hyakumanben Clinic, Kyoto, Japan; 7 Department of Human Environmental Design, Faculty of Health Science, Kio University, Kitakatsuragi-gun, Nara, Japan; 8 Kyoto Prefectural University of Medicine, Kyoto, Japan; Duke University, United States of America

## Abstract

Multiple chemical sensitivity (MCS) is characterized by somatic distress upon exposure to odors. Patients with MCS process odors differently from controls. This odor-processing may be associated with activation in the prefrontal area connecting to the anterior cingulate cortex, which has been suggested as an area of odorant-related activation in MCS patients. In this study, activation was defined as a significant increase in regional cerebral blood flow (rCBF) because of odorant stimulation. Using the well-designed card-type olfactory test kit, changes in rCBF in the prefrontal cortex (PFC) were investigated after olfactory stimulation with several different odorants. Near-infrared spectroscopic (NIRS) imaging was performed in 12 MCS patients and 11 controls. The olfactory stimulation test was continuously repeated 10 times. The study also included subjective assessment of physical and psychological status and the perception of irritating and hedonic odors. Significant changes in rCBF were observed in the PFC of MCS patients on both the right and left sides, as distinct from the center of the PFC, compared with controls. MCS patients adequately distinguished the non-odorant in 10 odor repetitions during the early stage of the olfactory stimulation test, but not in the late stage. In comparison to controls, autonomic perception and negative affectivity were poorer in MCS patients. These results suggest that prefrontal information processing associated with odor-processing neuronal circuits and memory and cognition processes from past experience of chemical exposure play significant roles in the pathology of this disorder.

## Introduction

Multiple chemical sensitivity (MCS) is a chronic acquired disorder characterized by non-specific and recurrent symptoms in multiple organ systems associated with exposure to low levels of odorous chemicals (e.g., organic solvents, pesticides, cleaning products, perfumes, environmental tobacco smoke or combustion products) [1]–[3]. The symptoms of MCS are reactions to previous chemical exposure that recur on subsequent exposure to the same or structurally unrelated chemicals at levels below those established as having harmful effects in the general population [2]. Patients with MCS report a variety of symptoms involving the central nervous system (CNS); respiratory, skin and mucosal irritation and gastrointestinal, musculoskeletal and cardiovascular problems. Other reported symptoms include fatigue, headaches, irritability, cognitive dysfunction, loss of concentration and memory, dizziness, anxiety, dyspnea, cough, skin irritation, dyspepsia, myalgia and many others [2], [4]. CNS-related symptoms such as headache, fatigue and cognitive deficits are especially frequent among MCS patients [5], [6].

Diagnosis of MCS can be difficult because of the inability to assess the causal relation between exposure and symptoms [3], [7]. No standardized objective measures for the identification of MCS and no precise definition of this disorder have been established. Therefore, most definitions of MCS are almost entirely qualitative, relying on subjective reports from patients and clinicians of distressing symptoms and environmental exposure. Some authors prefer the term idiopathic environmental intolerance to avoid the confusion of diagnosis and aetiology inherent in the term multiple chemical sensitivity [8], [9]. The symptoms of MCS may be related to specific psychiatric disorders rather than a toxicogenic or somatic source [2], [10]. However, in some cases, symptoms cannot be explained solely on a psychogenic basis.

Non-specific neural symptoms or dysautonomia may be evident in some MCS patients who exhibit high sensitivity to odors after exposure to small amounts of certain chemical substances [11]. One of the more plausible theories regarding the pathogenesis of MCS is that a chemical factor triggers a multi-organic response because of neurologic sensitization. This is plausible, given the interconnections between the olfactory system, limbic system and hypothalamus [12], [13]. However, no studies have confirmed this theory or any other neurologically-based mechanisms proposed with regard to the origin of MCS [14], [15].

Studies involving activation using positron emission tomography (PET) with several different odorants have indicated that patients with MCS process odors differently from controls. Regions of the brain engaged in odor processing (the amygdala, piriform cortex and insular cortex) are less activated in MCS patients than in controls; furthermore, an odorant-related increase in activation of the anterior cingulate cortex (ACC) and cuneus/pre-cuneus is observed [16]. Baseline regional cerebral blood flow (rCBF) in MCS patients was otherwise normal; abnormal patterns were observed only in response to odor signals. This pattern of activation in MCS may be a top-down regulation of odor response via the cingulate cortex. Furthermore, the results of challenge tests by exposure to odorous chemicals indicated neuro-cognitive impairment in MCS patients, and single photon-emission computed tomography brain dysfunction was found particularly in odor-processing areas, thereby suggesting a neurogenic origin of MCS [17]. In functional magnetic resonance imaging (fMRI) studies involving exposure to odorants, a strong signal-intensity reaction was seen in the limbic system of MCS patients [18]. This result suggests that fMRI analysis may be useful in the diagnosis of MCS. These studies provide useful pathophysiological information regarding the symptoms associated with MCS, enhancing our general understanding of this disorder. However, the use of these imaging modalities may put a physical or psychological burden on patients because of the risk of reactions with contrast agents, long testing periods and radiation exposure.

Near-infrared spectroscopy (NIRS) is an optical technique that provides a non-invasive measure of changes in haemoglobin and oxygenation in the human brain [19]. NIRS works on the principle that near-infrared light is absorbed by oxygenated (oxyHb) and deoxygenated (deoxyHb) haemoglobin (Hb), but not by other tissues. Although the spatial resolution of NIRS is inferior to that of other functional neuroimaging methodologies such as PET and fMRI, NIRS has the advantage of a high time resolution of <0.01 s and the feasibility of being performed under natural conditions [20]. Changes in blood flow and oxygenation in the brain are closely linked to neural activity. Changes in oxyHb concentration during tasks reflect neuronal activity because they correlate with evoked changes in rCBF [21]–[23]. When neurons become active, local blood flow to the relevant brain regions increases and oxygenated blood displaces deoxygenated blood. Measurement of oxyHb concentrations is most useful because changes in oxyHb are the most sensitive indicators of changes in rCBF among the three NIRS parameters (oxyHb, deoxyHb and totalHb) [24], [25].

Near-infrared rays sent out from the NIRS device can provide visual access to the cerebral cortex within approximately 20 mm from the scalp. An odorant-related increase in activation in the ACC has been observed in MCS patients [16]. The ACC is involved in adequate control of top-down or bottom-up modulation of stimuli and is connected to the prefrontal cortex (PFC) [26]. Therefore, evaluation of rCBF in the PFC using NIRS imaging may provide valuable information on specific activation due to odor stimulation in MCS patients. This may aid in defining and clarifying the pathology of this disorder. In this study, a simple test for diagnosing MCS was developed, and it involved the evaluation of changes in rCBF in the PFC of MCS patients.

## Methods

### Patients

MCS patients were diagnosed in the outpatient department for people with chemical sensitivities in the Hyakumanben Clinic (Outpatient Department of Sick House Syndrome). There are several case definitions for MCS, including those of Randolph in 1965 [27], Cullen in 1987 [28], Nethercott et al. in 1993 [29] and the MCS 1999 Consensus in the United States [30]. The most comprehensive and well-known case definition is the MCS 1999 Consensus [31]. Hence, MCS was diagnosed according to the 1999 consensus criteria [30] at the Hyakumanben Clinic between October 2009 and December 2011. Criteria were as follows: 1) Symptoms in multiple organ systems that were reproducibly triggered by exposure to low levels of multiple chemically unrelated and odorous chemicals. 2) Chronic symptoms (more than 1 year) that could be improved or resolved by removal of the incidents. The symptoms associated with MCS have similarities to those of chronic fatigue syndrome, fibromyalgia [32], [33] and psychological disorders [34]. Therefore, patients diagnosed with chronic fatigue syndrome or fibromyalgia syndrome were excluded from the study. In addition, patients suspected of having psychological disorders were examined by a qualified psychiatrist or practitioner of psychosomatic medicine, and those diagnosed with mental health disorders as per the Diagnostic and Statistical Manual of Mental Disorders IV or the International Classification of Diseases 10 were also excluded from the study. These criteria have been used in previous Japanese studies [35], [36]. Furthermore, patients who had hyperpiesia, hyperlipidemia, diabetes and allergic rhinitis were also excluded.

All MCS patients had been receiving treatment for MCS at this clinic. Recruitment for this study was conducted in the 3 months prior to the olfactory stimulation test using NIRS. The MCS condition of all patients was reconfirmed by the clinic physician on the occasion of the recruitment. Controls were recruited from the public and were selected to match patients by age and sex at the group level. The peripheral blood of all patients and controls was tested for the usual parameters (Blood cells, Hb, Ht, PL, T-BiL, TP, Alb, AST, ALT, γ-GT, ALP, LDH, ChE, AMY, CPK, BUN, Cre, GLU, HbA1c, LDL, HDL, TG, Na, Cl, K, Ca, CRP, RF, ANA, HBs, HCV, NK activity). Results of all haematological examinations were normal. Exclusion criteria for all patients and controls included smoker; drug or alcohol abuse; current use of antihypertensive medication, antihistamines or rheumatoid arthritis agents; pregnancy and severe nasal stuffiness.

The validated self-report Quick Environmental Exposure and Sensitivity Inventory (QEESI) [37] was utilized to confirm patient selection. For patients to be designated as chemically sensitive, high scores on the Chemical Intolerance (≥40), Other Intolerance (≥25) and Symptom Severity (≥40) scales are necessary [37]. In this study, MCS patients were included if they met or exceeded at least two of the three cut-off scores. Control patients were included if they met or exceeded one or none of the three cut-off scores.

This study was approved by the ethical committee for human research at the Hyakumanben Clinic (99642-61) and the Louis Pasteur Centre for Medical Research (LPC.11) and was performed according to the guidelines of the Declaration of Helsinki (1975). All patients provided written informed consent and received the equivalent of 5000 JPY for their participation. This study was conducted from November 2010 to March 2012.

### Olfactory stimulation

The card-type olfactory identification test kit (Open Essence; Wako Pure Chemical Industries, Ltd., Osaka, Japan) was used for the olfactory stimulation test. The capsuled odorant on the card is printed, folded and pressed flat. The cards are numbered and 12 kinds of odorants are included. These 12 odorants are the same as those used in the OSIT-J (Odor Stick Identification Test for the Japanese). Therefore, they are naturally compatible with the OSIT-J for Japanese patients with olfactory disturbance [38]. Significant correlations were found among the score for Open Essence, the average recognition threshold of the T&T olfactometer (Japanese standard olfactory test kit) and OSIT-J scores [39]. The examination time for Open Essence is the shortest among these three tests. Therefore, very little time is required for this examination. This kit is single-use and does not require a rubbing tool. This guarantees total cleanliness and no contamination of odorants [39]. In the olfactory stimulation test used in this study, the use of odorants that were harmless to the test patients and general population and were commonly perceived during ordinary daily activities was required. Therefore, of the 12 odorants, four (mandarin orange, Japanese cypress, menthol and perfume) were used in this study. Perception of these odors was accomplished by placing the test card at a distance of approximately 30 mm from the noses of both MCS patients and controls.

### Experimental procedure

Interviews were conducted just prior to the olfactory stimulation test and the assessments of health and nasal symptoms. The test room was maintained at a temperature of approximately 22°C. Patients sat in a comfortable chair in the room. They remained in the test room long enough to feel comfortable before being exposed to the odorants. During the experiments, the patients closed their eyes and slowly repeated the Japanese alphabet in an undertone to establish a stable rCBF prior to the olfactory stimulation. And they continued to close their eyes and stopped to repeat Japanese alphabet during olfactory stimulation. The questionnaire on irritating and hedonic scale was completed immediately after the olfactory stimulation. After that, they again closed their eyes and slowly repeated the Japanese alphabet in an undertone to establish a stable rCBF prior to the next olfactory stimulation. Irritation was evaluated on a visual analogue scale, with responses ranging from “not at all” to “strong”. Hedonic responses were rated on a 5-point Likert scale ranging from comfort (1) to discomfort (5). Olfactory stimulation was performed with 10 repetitions of 30 s each after a rest period to establish the baseline level, followed by a 10-s period of olfactory stimulation and a 30-s rest period for stabilization of the olfactory mechanism. The 10 repetitions were performed continuously, and the time between tasks was 60 s. Olfactory stimuli were offered in the following order: mandarin orange (MO), perfume (Pf), non-odorant (NO), Japanese cypress (JC), menthol (Mt), Pf, JC, NO, Mt and MO. The orders of ten repetitions (1 to 10) were presented as follows: MO (1), Pf (2), NO (3), JC (4), Mt (5), Pf (6), JC (7), NO (8), Mt (9) and MO (10).

### NIRS data acquisition

**Figure 1:**
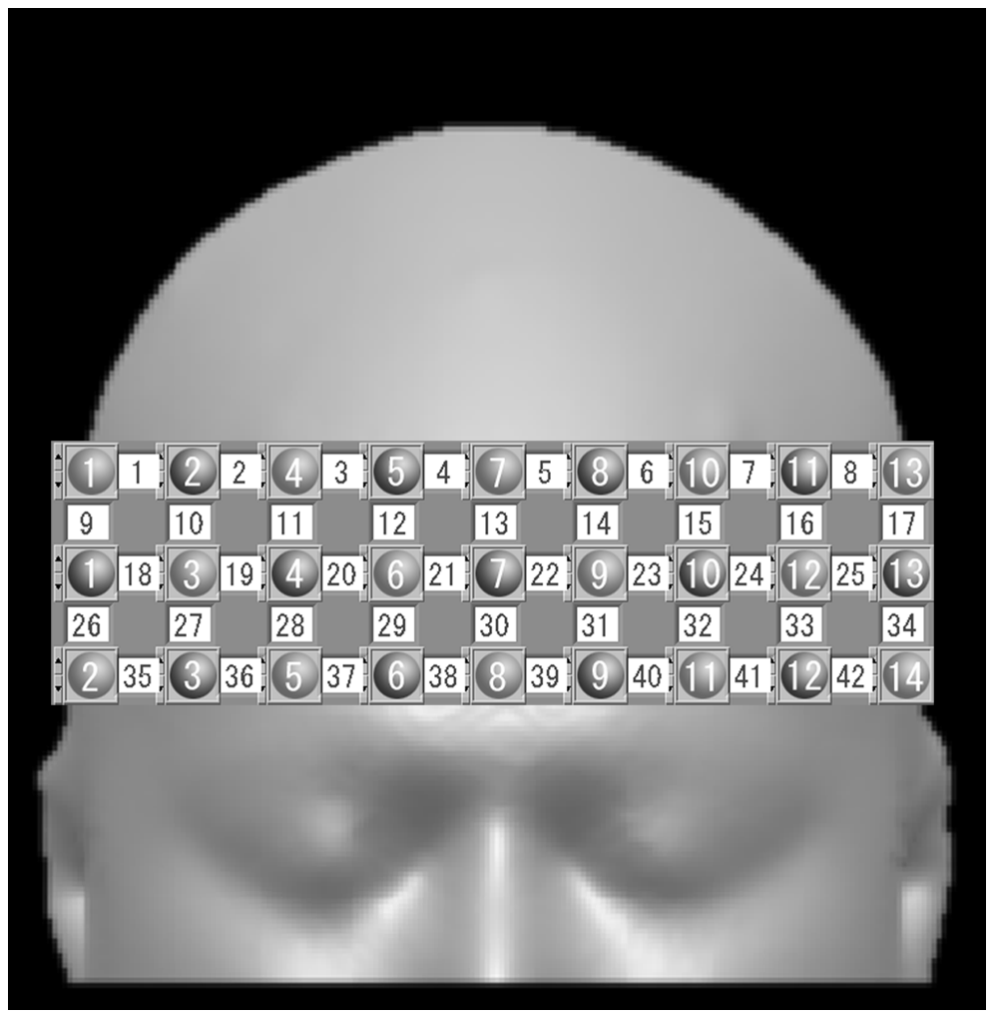
Experimental setting and NIRS channel orientation. Detectors and illuminators are shown as gray circles (1 to 14). Channels are shown as white squares (1 to 42). The international 10–20 standard positions and other positional information are indicated. One holder with 42 NIRS channels was set on the PFC of each patient so that the midpoint of channels 38 and 39 corresponded to the intersection point of the F7, F8 and Fz of the international 10–20 system and channels 35 to 38 and 39 to 42 aligned with F8 and F7, respectively.

Changes in Hb concentration in the PFC were measured using the functional NIRS topography system OMM-3000 Optical Multi-channel Monitor (Shimadzu Corporation, Kyoto, Japan), which uses near-infrared light with wavelengths of 780, 805 and 830 nm. Pairs of illuminators and detectors were set 3 cm apart in a 3×9 lattice pattern to form 42 channels through a holder set in the PFC (Figure 1). Changes in concentration of oxyHb, deoxyHb and totalHb were recorded every 130 ms using the NIRS system. However, only oxyHb was analysed because these changes are the most sensitive indicators of changes in rCBF and provide the strongest correlation with blood oxygenation level-dependent signals among the three NIRS parameters [24], [25]. Optical data were analysed on the basis of the modified Beer–Lambert Law and signals reflecting the oxyHb concentration changes in an arbitrary unit were calculated (millimolar–millimetre) [40].

### Questionnaire on physical and psychological status

Patients completed a self-report questionnaire for the assessment of physical and psychological parameters as follows. Affective reactions to and behavioural disruptions in daily activities from odorous/pungent environmental chemicals were assessed using the Chemical Sensitivity Scale for Sensory Hyper-reactivity (CSS-SHR) [41]. Somatosensory amplification was assessed using the Somato-Sensory Amplification Scale (SSAS) [42]. Somatosensory amplification refers to a tendency to experience physical sensations as intense, noxious and disturbing. The anxiety-related version of the Autonomic Perception Questionnaire (APQ) was used to evaluate attentiveness to physical responses in anxiety-provoking situations [43]. The Tellegen Absorption Scale (TAS) was used to measure imaginative involvement and openness to experience [44]. Repressive coping was assessed by the Marlowe–Crowne Social Desirability Scale [45] and the Taylor Manifest Anxiety Scale (TMAS) [46]. The Negative Affectivity Scale (NAS) was used to evaluate the tendency to experience and report negative emotions, including anxiety, guilt, hostility and depression, with a low negative affect reflecting a state of calmness [47]. Lastly, alexithymia was assessed using the Toronto Alexithymia Scale (TAS-20) [48]. Alexithymia can be evaluated using both the total score and the scores of the three subscales, which assess difficulties in identifying feelings (DIF), difficulties in describing feelings (DDF) and externally-oriented thinking (EOT). Information about these questionnaires and the physical and psychological scales was provided by the Danish Research Centre for Chemical Sensitivities.

### Statistical analyses

Changes in oxyHb concentration are the best indicators of changes in rCBF and brain activity. Therefore, oxyHb levels during the olfactory stimulation were compared with oxyHb levels during the pre-rest period as a baseline level in each channel for evaluating the effects on brain activity of olfactory stimulation. Because raw data of NIRS provided only relative values and could not be averaged directly across patients or compared among channels, raw data from each channel were converted into z-scores [49]–[51]. The z-score was calculated using the mean value and standard deviation of oxyHb changes during the pre-rest period. Consequently, mean values and standard deviations during the pre-rest period were respectively changed into z-scores 0 (mean value) and 1 (standard deviation) for every channel. The *t*-test was used to compare brain activity from NIRS imaging for each channel between cases and controls. The non-parametric Mann–Whitney U test was utilized for analysis of the results of the questionnaire administered after the olfactory stimulation test to determine differences between MCS patients and controls. The *t*-test was applied for analysis of the results of the physical and psychological scales to determine differences between MCS patients and controls at baseline. All data analyses were performed using the SPSS statistics software, version 21.

## Results

### Participants

Participants were 16 MCS patients (age, 44–65 years; mean, 53.5±7.0 years; 1 male, 15 females) and 17 controls (age, 39–62 years; mean, 50.2±8.4 years; 1 male, 16 females). Twelve non-smoking MCS patients (age, 47–65 years; mean, 55.1±6.8 years; all females) and 11 non-smoking controls (age, 39–61 years; mean, 48.0±8.0 years; 1 male, 10 females) passed all criteria and were included in the analyses. All MCS patients tried to avoid exposure to odorous chemicals as much as possible. Occupational histories showed that three MCS patients were clerical employees (hospital, office and retail store) and nine were homemakers or pensioners whose previous occupations included clerical employee (museum or office), teacher, endoscopic operator, fabric tinter and supermarket baker. Eight controls also tried to avoid exposure to odorous chemicals as much as possible. Of them, occupations of six included teacher, office worker, tester of ceramic parts and voluntary worker in an environmental laboratory, and of the remaining two, one was a pensioner and other was a homemaker. Three controls consciously did not try to avoid exposure to odorous chemicals and their occupations were office worker, child welfare volunteer and voluntary worker in an environmental laboratory.

### NIRS imaging and subjective evaluation to odors

Results of the *t*-test in terms of the average of all channels (1 to 42) comparing z scores for oxyHb concentrations between MCS patients and controls are shown in Table 1. In the olfactory stimulation involving MO (1), which was conducted first, increases in rCBF levels in the PFC were observed in both MCS patients and controls. The difference in rCBF level between these groups was not significant. Because MO (1) was the first test, the patients may not have had the chance to get used to the olfactory stimulation test. Therefore, this response may have been caused by affective tension. After the first test, no increases in rCBF level were observed in controls, and rCBF levels remained stable until the end of the test involving MO (10).

**Table 1:** Results of the *t*-test in terms of average values for all channels (1 to 42) comparing z scores for oxyHb between MCS patients and controls.

Test	MCS (*n* = 12)	Controls (*n* = 11)	*p* value
MO (1)	0.52 (1.54)	0.47 (0.96)	0.574
Pf (2)	0.55 (1.78)	0.07 (1.00)	<0.001*
NO (3)	0.22 (1.00)	0.17 (0.73)	0.343
JC (4)	0.71 (1.63)	0.03 (1.02)	<0.001*
Mt (5)	0.41 (1.44)	0.09 (0.96)	<0.001*
Pf (6)	0.58 (1.46)	0.08 (0.80)	<0.001*
JC (7)	0.71 (2.09)	0.26 (1.15)	<0.001*
NO (8)	0.45 (1.10)	0.09 (0.87)	<0.001*
Mt (9)	0.39 (2.02)	−0.16 (0.85)	<0.001*
MO (10)	0.77 (1.70)	0.06 (0.99)	<0.001*

Values are expressed as means (± standard deviations).

*Significant at *p*<0.05.

Abbreviations: MO, mandarin orange; Pf, perfume; NO, non-odorant; JC, Japanese cypress; Mt, menthol. Numbers in parentheses in column 1 indicate the order of the 10 repetitions (1 to 10).

**Figure 2:**
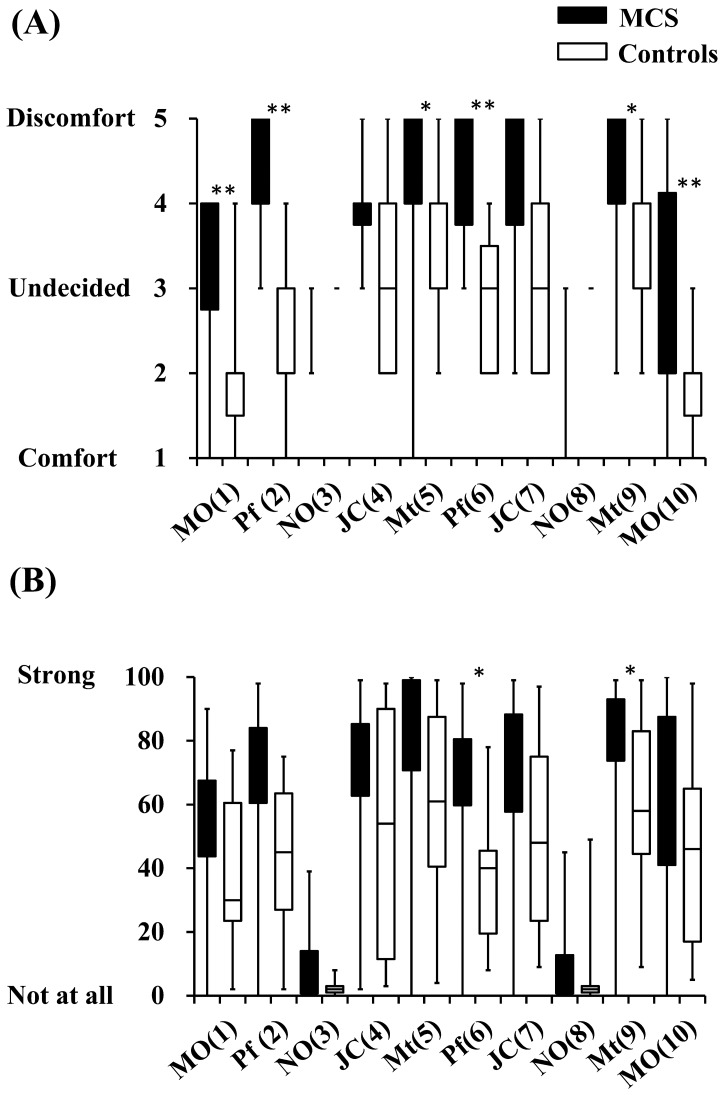
Ratings of hedonic (A) and irritating (B) odours by MCS patients (*n* = 12) and controls (*n* = 11) after the olfactory stimulation. Abbreviations: MO: mandarin orange, Pf: perfume, NO: non-odorant, JC: Japanese cypress, Mt: menthol. Numbers in parentheses indicate orders of ten repetitions (1 to 10). Statistically significant differences between groups are indicated. ^*^ *p*<0.05, ^**^ *p*<0.01.

Increases in rCBF levels in MCS patients were suppressed during the olfactory stimulation involving NO (3) on the third repetition. Responses in the PFC were normal; the difference between MCS patients and controls was not significant. However, on the eighth repetition involving NO (8), PFC activation was observed in MCS patients. This difference between MCS patients and controls was significant (*p*<0.001). This result suggested that the olfactory system in MCS patients adequately distinguished the non-odorant among the 10 odorant repetitions during the early stage of the olfactory stimulation test. However, this result also suggested that the olfactory system in MCS patients could not adequately process odors in the late stage of the olfactory stimulation test. Table 2 shows the correlation coefficient between rCBF after the first and second exposure of the same odor in terms of z scores for all channels (1 to 42). Comparing the rCBF between first and second exposures revealed significant correlations in both MCS patients and controls. However, the correlation coefficients of MCS patients were lower overall than those of controls. In the subjective evaluation, both MCS patients and controls responded “not at all” on the irritation scale and “undecided” on the hedonic scale for NO (Figure 2). However, NIRS imaging revealed that the CNS of MCS patients may have been confused in the late stage of the olfactory stimulation test.

**Table 2:** Correlation coefficient (r) between rCBF after the first and second exposures to the odor in terms of z scores for all channels (1 to 42).

Odorant	MCS (*n* = 12)		Controls (*n* = 11)	
	*r*	*p* value	*r*	*p* value
MO	0.418	<0.001*	0.352	<0.001*
Pf	0.166	<0.001*	0.649	<0.001*
NO	0.395	<0.001*	0.526	<0.001*
JC	0.268	<0.001*	0.478	<0.001*
Mt	0.372	<0.001*	0.407	<0.001*

Values are expressed as Pearson product-moment correlation coefficients.

*Significant at *p*<0.05.

Abbreviations: MO, mandarin orange; Pf, perfume; NO, non-odorant; JC, Japanese cypress; Mt, menthol.

**Figure 3:**
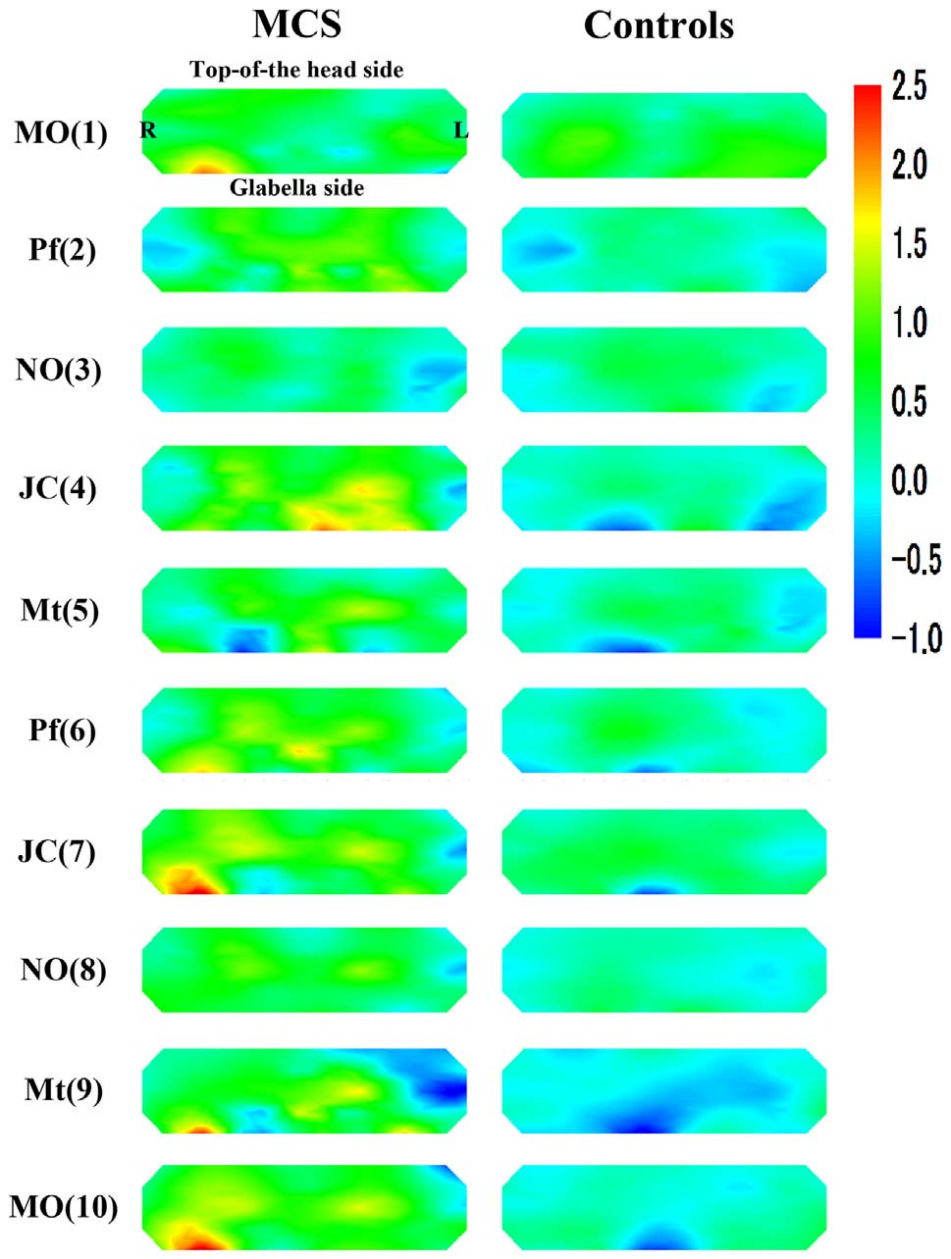
Topographical maps of average z scores for oxyHb in MCS patients (*n* = 12) and controls (*n* = 11). Abbreviations: MO: mandarin orange, Pf: perfume, NO: non-odorant, JC: Japanese cypress, Mt: menthol. Numbers in parentheses indicate the order of the 10 repetitions (1 to 10).

**Figure 4:**
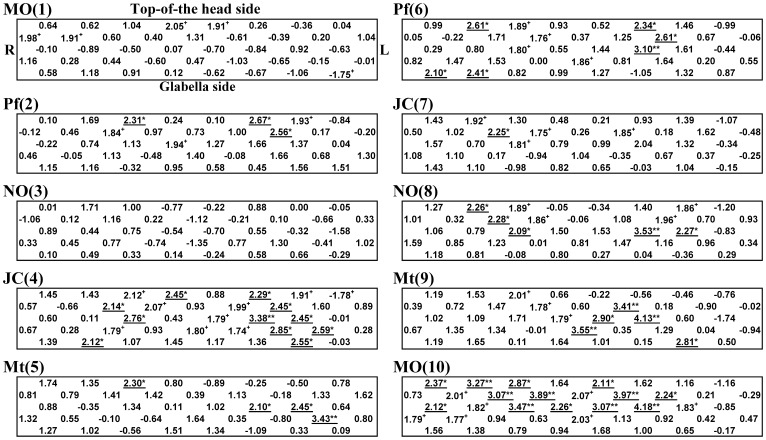
Average *t* value of each channel comparing z scores for oxyHb between MCS patients (*n* = 12) and controls (*n* = 11). Statistically significant differences between groups are indicated as underlined values. ^*^ *p*<0.05, ^**^ *p*<0.01. Significant tendencies are indicated: ^+^ *p*<0.10.

Figure 3 provides topographical maps of average z scores for oxyHb in MCS patients and controls. Figure 4 shows average *t* values for each channel comparing z scores for oxyHb between MCS patients and controls. Significant activation in the PFC was observed for MCS patients on both the right and left sides (as distinct from the center of the PFC) compared with controls. Activation was defined as a significant increase in rCBF due to olfactory stimulation. These activations were stronger in the test for JC (4) on the fourth repetition and that for Pf (6) on the sixth repetition. In the tests for MO (1), Pf (6), JC (7), Mt (9) and MO (10), strong increases in rCBF were observed in the bottom right of PFC in MCS patients (Figure 3). However, no significant differences were found in the results of tests other than those for Pf (6) between MCS patients and controls (Figure 4).

The results of subjective evaluation using the hedonic scale indicated that scores for MCS patients were significantly higher than those for controls, except JC scores. Scores for MO were lower than those for the other odorants in both MCS patients and controls. The results of subjective evaluation using the irritation scale indicated that Pf (6) and Mt (9) scores for MCS patients were significantly higher than those for controls. However, no differences were found for other odors. Large ranges of scores in controls were thought to be causally related to the results.

### Physical and psychological measurements

Table 3 shows the results of the *t*-test for the physical and psychological scales. CSS-SHR scores were significantly higher for MCS patients than for controls (*p*<0.001). Therefore, chemical sensitivity in MCS patients was demonstrated not only by the results of the QEESI but also by those of the CSS-SHR scale. In the psychological evaluations, APQ (*p*<0.001), NAS (*p* = 0.005) and TAS-20 DIF (*p*<0.001) scores were significantly higher for MCS patients than for controls. However, no significant differences were observed in the SSAS, TAS, MCSD, TMAS, TAS-20 total, TAS-20 DDF and TAS-20 EOT scores.

**Table 3:** Results of the *t*-test for the physical and psychological scales.

Scales	MCS (*n* = 12)	Controls (*n* = 11)	*p* value
QEESI (CI)	79.6 (18.6)	26.6 (21.0)	<0.001*
QEESI (OI)	34.7 (24.0)	4.0 (5.1)	<0.001*
QEESI (SS)	63.1 (14.9)	8.4 (5.8)	<0.001*
CSS-SHR^a^	49.7 (3.7)	34.0 (8.5)	<0.001*
SSAS^a^	33.1 (5.5)	30.1 (7.9)	0.313
APQ^a^	141.8 (38.8)	82.0 (28.9)	<0.001*
TAS^a^	12.5 (7.1)	8.6 (7.0)	0.227
MCSD^a^	17.6 (3.1)	17.3 (5.1)	0.894
TMAS^a^	11.2 (3.4)	8.6 (2.8)	0.073
NAS^a^	41.1 (12.7)	26.3 (6.1)	0.005*
TAS-20 total^a^	47.2 (11.4)	40.5 (6.3)	0.111
TAS-20 DIF^a^	14.0 (3.3)	9.2 (2.0)	<0.001*
TAS-20 DDF^a^	13.4 (4.4)	11.0 (2.9)	0.168
TAS-20 EOT^a^	19.8 (6.3)	20.3 (4.7)	0.845

Values are expressed as means (± standard deviations).

*Significant at *p*<0.05.

a Because of missing values, *t*-test results included the following numbers of patients. MCS and control: CSS-SHR, MUSS, APQ, TMAS, MCSD, TAS, TAS-20, TAS-20 DIF, TAS-20 DDF and TAS-20 EOT, *n* = 11 and *n* = 10; SSAS, CHS, CNSS and CSAS, *n* = 11 and *n* = 11; NAS, *n* = 10 and *n* = 11.

Abbreviations: CI, chemical intolerance; OI, other intolerance; SS, symptom severity.

## Discussion

Responses in the PFC in MCS patients were normal for NO (3), that is, the third repetition providing the non-odorant condition. The difference in response to this condition was not significant between MCS patients and controls. Activation of the PFC in MCS patients was evident for NO (8), that is, the eighth repetition providing the non-odorant condition. PFC activation for MO (1) in MCS patients was higher than that for MO (10). These results suggest that the olfactory system in MCS patients could not adequately process odors in the late stage of the olfactory stimulation test. However, no PFC activation was observed for these odors in controls. In addition, rCBF remained stable until the final repetition involving MO (10) in controls.

Inherent connections of the frontal lobe form vital feed-forward and feedback circuits from the center of prefrontal information processing. The extensive connections in the PFC are linked with distant and broadly dispersed parts of the association and limbic cortices. Prefrontal interconnections with the amygdala, hypothalamus, midbrain and pons represent important subcortical linkages of the extended prefrontal neural system. These are likely to integrate higher-order brain functions mediated by the PFC with more developmentally fundamental brain activities such as emotional, visceral or autonomic functions [26], [52], [53]. Therefore, the center of the PFC depends significantly on emotional linkages with deeper brain structures related to control of pleasure, pain, anger, rage, panic and aggression. On the basis of this information, we postulate that prefrontal information processing in MCS patients was activated by an emotional response to repeated olfactory stimulation in the late stage of the test and that the processing system in the PFC could not properly respond despite differences in subjective reports about the odors. These results suggest that this response may be characteristic of MCS patients. Activation of the PFC may therefore have occurred during the olfactory stimulation test using odorants ordinarily encountered in daily activities.

This study specifically demonstrated activation in the PFC on both the right and left sides, as distinct from the center of the PFC, in MCS patients compared with that in controls during olfactory stimulation tests. Activation was observed in the early stage of the olfactory stimulation tests, when the odor processing systems of MCS patients were stable. In a previous study, patients with MCS processed odors differently from controls, and an odorant-related increase in activation of the ACC and cuneus–pre-cuneus was observed [16]. The dorsal part of the ACC is connected with the PFC and parietal cortex as well as the motor system and frontal eye fields. Therefore, it is essential for processing top-down and bottom-up stimuli and assigning appropriate control to other areas in the brain. In contrast, the ventral part of the ACC is connected with the amygdala, nucleus accumbens, hypothalamus and anterior insula and is involved in assessing the salience of emotion and motivational information [26], [54]–[56]. MCS occurs when individuals are first sensitized via an initial exposure to a certain amount of chemicals or repeated exposure to small amounts of chemicals. Upon re-exposure, individuals become increasingly sensitized, and often the effect spreads and they become bothered by many additional chemicals [1]. In this study, nine MCS patients had episodes of initial exposure to chemicals that triggered the first symptoms. These included use of organic solvents, pesticides or incense in the workplace, use of pesticides or diesel machines in the neighborhood or use of pesticides indoors. Three patients had episodes of repeated exposure to solvents emitted from a neighboring industrial plant or paint store or fragrances or tobacco smoke emitted around the neighborhood. MCS patients complained about a chemical sensitive condition thereafter. We suggest that these exposure events were stored as memories in the PFC through olfactory nerve circuits, causing various physical or psychological responses such as emotional, visceral or autonomic reactions during processing of top-down stimuli in later life when they exposure to odorants. The psychological evaluations in our study indicated that scores in MCS patients were significantly higher than those in controls on the APQ, NAS and TAS-20 DIF scales. These results also support the theory of response regulation by memory in the PFC described above. NIRS imaging in combination with the olfactory stimulation test may therefore be valuable for objective evaluation and identification of patients with MCS.

Several studies have reported characteristic changes in the odor-processing region of the brain due to olfactory stimulation in MCS patients [16]–[18], [57]. However, this is the first case–control study to evaluate changes in rCBF in the PFC using NIRS imaging during olfactory stimulation by odorants in MCS patients. Significant differences were found between MCS patients and controls. Further research regarding odor processing, stimulus detection, cognition, provoking memory and information communication between the PFC, ACC and olfactory nervous center during olfactory stimulation in MCS patients is required.

There are some possible limitations in the present study. First, the small sample size makes the results vulnerable to selection bias. This could be alleviated by including a larger study population. This is the first case–control study evaluating changes in rCBF in the PFC using NIRS imaging during olfactory stimulation in MCS patients. Activation in the PFC of MCS patients may be supported by a similar finding observed in the ACC in a previous study [16]. A follow-up study for MCS patients for comparison with symptom improvement in practice would also provide valuable information. A second limitation of this study is the selection of the study group. No standardized objective measures to identify and define MCS have been established. Therefore, most definitions of MCS are almost entirely qualitative, relying on subjective reports of distressing symptoms and environmental exposure from patients and clinicians. Several individuals with self-reported MCS symptoms were excluded, at the discretion of the clinic physician, because of mental disorders or allergic symptoms.

In conclusions, despite the small sample size, this experimental study identified activation in the PFC due to olfactory stimulation in MCS patients. The results indicated that NIRS imaging is a valuable method for the objective evaluation of MCS. In addition, the results suggest that prefrontal information processing associated with the odor-processing neuronal circuits and memory and cognition processing from past experience of chemical exposure may play significant roles in the pathology of this disorder.
